# The Role of Cross-Sectional Imaging in the Diagnosis of Agenesis of the Dorsal Pancreas: A Case Series

**DOI:** 10.7759/cureus.40930

**Published:** 2023-06-25

**Authors:** Nivedita Jha, Umakant Prasad, Deepak Kumar, Ruchi Gupta, Ashutosh Jha, Sanjay K Suman

**Affiliations:** 1 Radiodiagnosis, Indira Gandhi Institute of Medical Sciences, Patna, IND

**Keywords:** dorsal agenesis of the pancreas, pseudoagenesis of pancreas, pancreatitis, diabetes mellitus, mrcp

## Abstract

Background

Agenesis of the dorsal pancreas is a rare entity and is caused due to the defective embryological development of the pancreas. The clinical manifestations may range from diabetes mellitus, pancreatitis, and abdominal pain to no symptoms at all. We here present a case series of 10 cases with complete or partial agenesis of the dorsal pancreas.

Objectives

To correlate the clinical symptoms in the patients with the dorsal agenesis of the pancreas, to study any biochemical abnormality present with the dorsal agenesis of the pancreas, and to look for other coexistent finding in the patients.

Results

We observed that out of 10 patients, six were males and four were females. Four had symptoms related to the pancreas and six were discovered incidentally. Diabetes mellitus was present in five patients, seven patients had pain in the abdomen, and jaundice was seen in three patients. Out of 10 patients, four had complete agenesis and six had partial agenesis of the dorsal pancreas.

Conclusions

We conclude that the diagnosis of this rare entity and establishing its association with clinical conditions like diabetes mellitus and non-specific abdomen pain with the aid of cross-sectional imaging helps in the better evaluation and management of the patients.

## Introduction

Dorsal pancreatic agenesis is a rare congenital disorder characterized by abnormal embryogenesis and is compatible with life whereas complete agenesis of the pancreas or ventral agenesis is incompatible with life [1]. Dorsal pancreatic agenesis can be asymptomatic or cause epigastric discomfort, hyperglycemia, and acute or chronic pancreatitis [2]. The first case of dorsal pancreatic agenesis was noticed during an autopsy in 1911 [3]. Less than 100 cases have been reported in the literature since then [4]. Increased use of cross-sectional imaging, including CT and MRI, may be the cause of an increase in the detection of dorsal agenesis of the pancreas.

To the best of our knowledge, this study is the first to comprise a series of 10 cases of agenesis of the dorsal pancreas.

## Materials and methods

We conducted both a retrospective review and prospective observational study on 10 patients after getting approval from the Institutional Ethics Committee (IEC), Indira Gandhi Institute of Medical Sciences, Patna in the Department of Radiodiagnosis of our institute, from 1st January 2021 to 31st December 2022. The patients presented with either non-specific abdomen pain and early onset of diabetes or they were detected incidentally during the evaluation of other primary pathologies. The routine blood investigations, including complete blood count, serum bilirubin, blood glucose, serum amylase, and lipase, were collected. Informed consent was taken from the patients. Contrast-enhanced computed tomography (CECT) of the abdomen was done for all 10 patients and six patients underwent MRI after overnight fasting. The CT and MRI images were assessed for the dorsal agenesis of the pancreas, whether it was partial or complete, with dependent stomach and intestine signs, and other imaging findings. Ductal anatomy was specifically evaluated in the MRI images.

Inclusion and exclusion criteria

Inclusion criteria included known and incidental cases of dorsal agenesis of the pancreas of any age group and both sexes (male and female).

Exclusion criteria included contraindication to MRI, e.g., claustrophobia, cochlear implant, and pacemaker, and patients who did not gave consent.

Equipment

The multidetector CECT scans were done on Toshiba Aquilion XL 128 slice multidetector computed tomography (MDCT) machine (Toshiba, Tokyo, Japan) at 5 mm collimation, 0.5 mm reconstruction interval, 0.5 second gantry rotation time, 120 kV, and 500 mAs. The contrast agent used was iohexol (Omnipaque 350) and the dose used was 1-1.5 mL/kg body weight. The images covered the area from the diaphragm above to the pubic symphysis below. After the pre-contrast images, scanning was done 70 seconds after the administration of IV contrast (portal venous phase).

MRI was done on a 1.5 Tesla superconducting machine Optima MR360 (GE HealthCare, Chicago, IL). The sequences taken were axial T2-weighted imaging (T2WI), axial T2-weighted fat-suppressed (T2FS), axial T1-weighted fat-suppressed (T1FS), axial diffusion-weighted imaging (DWI), coronal T2WI, axial three-dimensional magnetic resonance cholangiopancreatography (MRCP), and coronal fat-suppressed fast imaging employing steady-state acquisition (FIESTA). Intravenous or local contrast was not used.

Study design

This was a retrospective review and prospective observational study.

Statistical analysis

The data were transferred to a Microsoft Excel 2010 sheet (Microsoft Corporation, Redmond, WA) and statistical analysis was done using IBM SPSS for Windows version 22.0 (IBM Corp., Armonk, NY).

## Results

We conducted a retrospective review and prospective observational study and collected records regarding the age, sex, chief complaints, relevant history, routine investigations, and imaging findings of 10 patients. CT scan was done in all 10 patients and out of those patients, six underwent MRI. Six patients were males and four patients were females. The mean age of the patients was 44.5 years. The minimum age at presentation was 19 years and the maximum age was 74 years. Table 1 shows the age, gender, presenting complaints, relevant history, and imaging findings of all 10 patients.

**Table 1:** Chief presentation, relevant history, and imaging findings of 10 patients with agenesis of the dorsal pancreas. M: male; F: female; CBD: common bile duct.

Serial No.	Age	Sex	Chief presentation	Relevant history	Types of dorsal agenesis	Other imaging findings
1	55 years	M	Follow-up for bilateral nephrolithiasis	Pain in the abdomen, diabetes mellitus, jaundice	Partial	Duodenal diverticulum with compression and dilatation of CBD, Lemmel syndrome, dilated portal vein, bilateral hydroureteronephrosis with renal calculi
2	19 years	F	Diabetes mellitus	Jaundice	Complete	-
3	40 years	M	Acute pancreatitis	Pain in the abdomen	Partial	Cholelithiasis
4	40 years	F	Work-up for carcinoma ovary	-	Partial	Solid-cystic ovarian mass with ascites and peritoneal dissemination and duodenal diverticulum
5	31 years	M	Trauma	Pain in the abdomen	Partial	Splenic laceration with active extravasation of contrast
6	41 years	F	Work-up for carcinoma breast	Diabetes mellitus	Complete	Liver metastases
7	74 years	M	Work-up for carcinoma pancreas	Pain in the abdomen	Partial	Soft tissue mass in the body of the pancreas with calcific foci in the head region, chronic pancreatitis, and carcinoma pancreas
8	50 years	F	Generalized body swelling	Pain in the abdomen, jaundice	Partial	Duodenal diverticulum, ascites
9	45 years	M	Chronic pancreatitis	Pain in the abdomen, diabetes mellitus	Complete	Calcific foci in visualized head and uncinate process of the pancreas
10	50 years	M	Chronic pain abdomen	Diabetes mellitus	Complete	-

**Figure 1:**
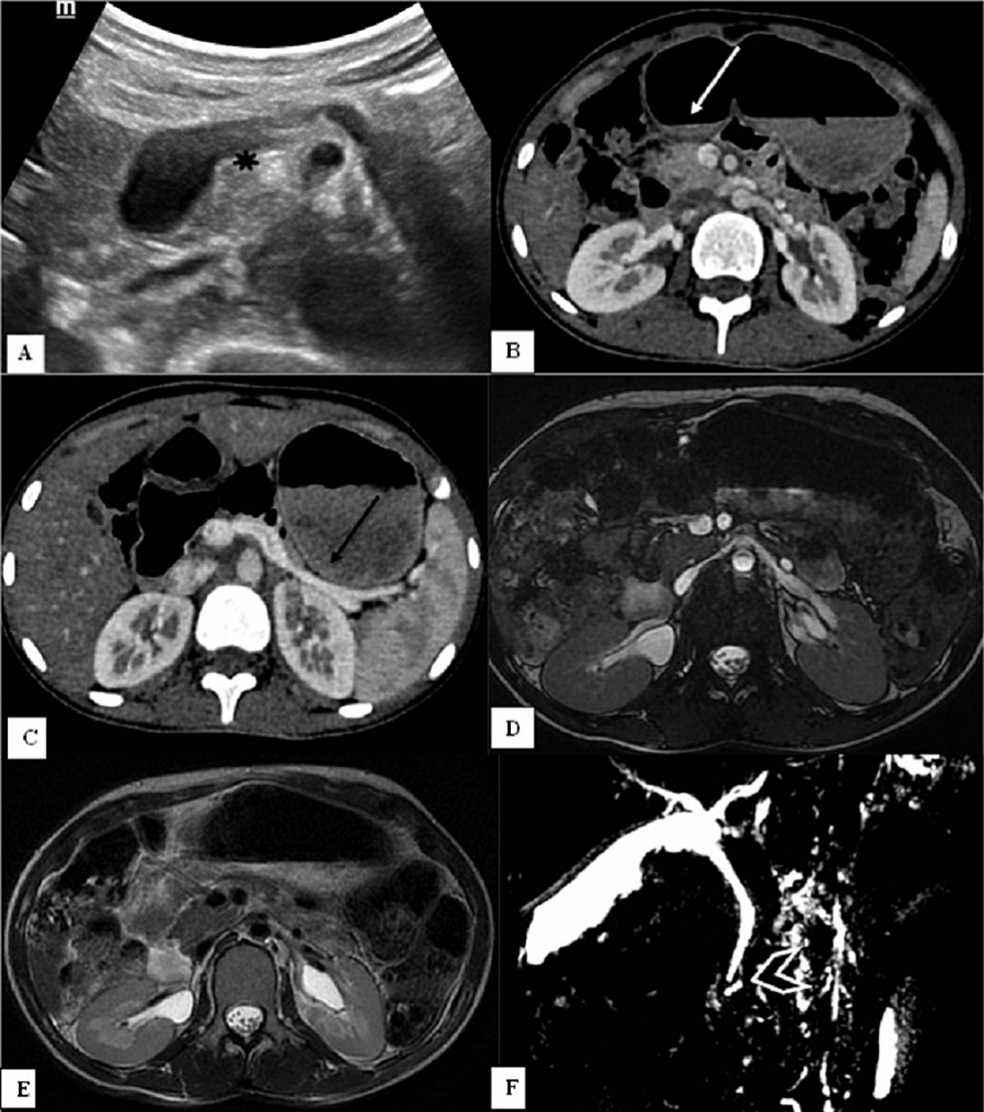
Complete dorsal agenesis of the pancreas. Transabdominal ultrasonography of a 19-year-old girl showing visualization of only the uncinate process and part of the head of the pancreas (A, black star). On the axial section, contrast-enhanced computed tomography scan images show the head of the pancreas (B, white arrow) but the neck, body, and tail of the pancreas are not visualized and potential space is filled with stomach and intestinal loops (C, black arrow). Magnetic resonance imaging axial fast imaging employing steady-state acquisition (FIESTA) (D) and axial T2-weighted image (E) showing the absence of body and tail of pancreas and computed tomography equivalent of the dependent stomach and dependent intestine sign. Magnetic resonance cholangiopancreatography (MRCP) (F) demonstrates a short major pancreatic duct, i.e., the duct of Wirsung (chevron arrow) and dorsal duct is not visualized.

**Figure 2:**
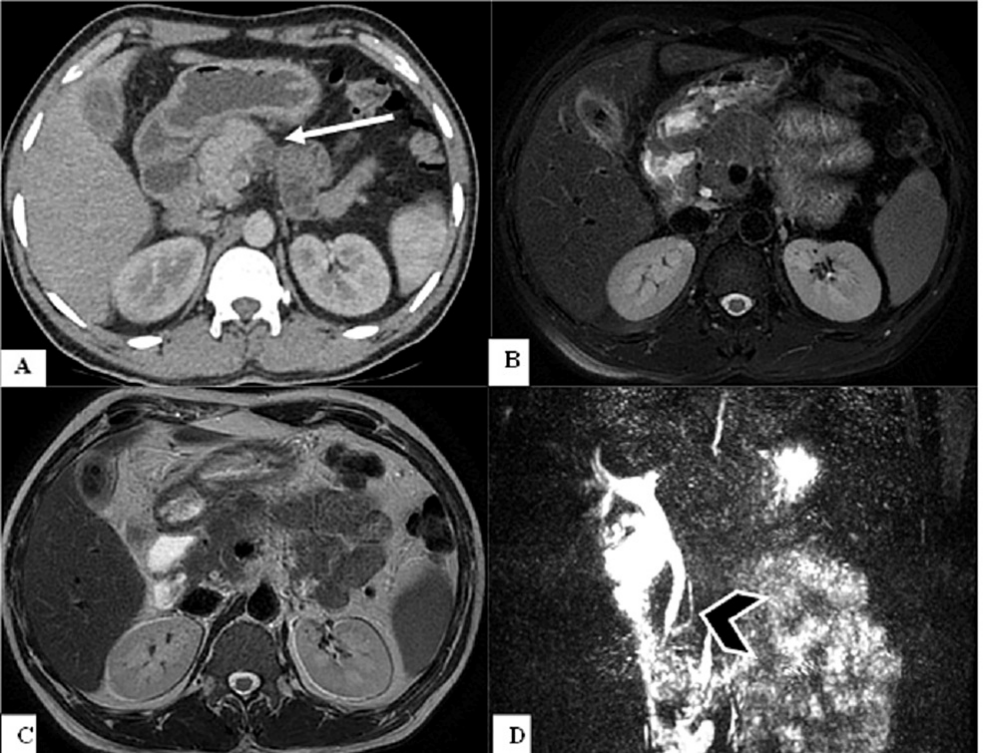
Acute pancreatitis in a case of partial agenesis of the dorsal pancreas. Contrast-enhanced computed tomography of the abdomen (A) showing heterogeneity in the visualized part of the head (white arrow). Axial T2 fat-suppressed image (B) and axial T2-weighted imaging (C) showing non-visualization of part of the body and tail of the pancreas with mild surrounding fat stranding. Magnetic resonance cholangiopancreatography (D) showing the short main pancreatic duct (chevron arrow). Serum amylase and lipase levels of the patient were raised.

**Figure 3:**
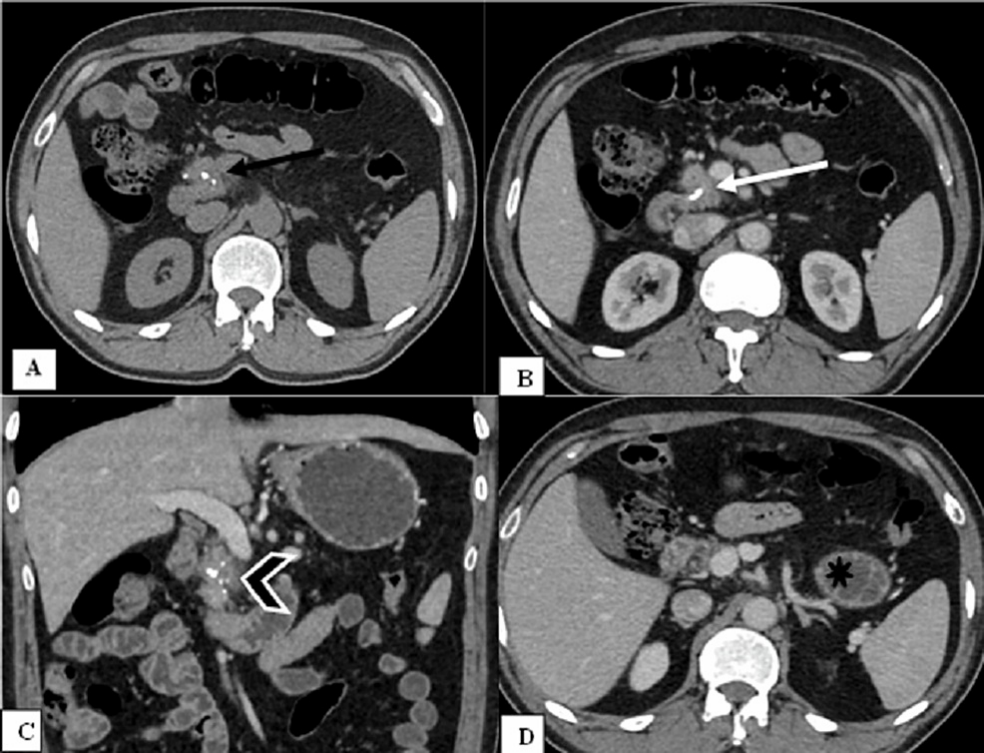
Agenesis of the dorsal pancreas with chronic calcific pancreatitis. Non-contrast computed tomography (A, black arrow) and contrast-enhanced computed tomography (B, white arrow) axial and coronal (C, chevron arrow) images of the abdomen show a slightly atrophic head of the pancreas with specks of calcification and non-visualization of the body and the tail of the pancreas. The dependent intestine sign (black star) can be seen in the subsequent image (D).

**Figure 4:**
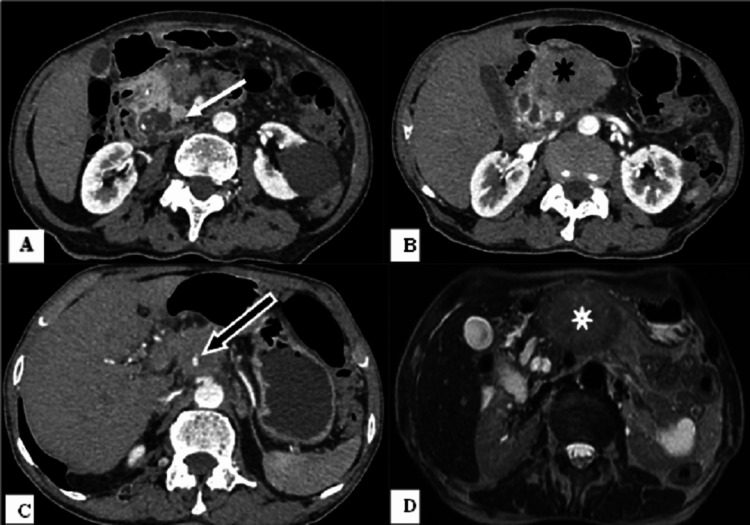
Chronic pancreatitis and carcinoma pancreas in a case of partial agenesis of the dorsal pancreas. Contrast-enhanced computed tomography of the abdomen (A) showing calcific foci with dilated duct (white arrow) in the head of the pancreas. An ill-defined heterogeneously hypoenhancing mass (B, black star) is seen in the visualized region of the body of the pancreas encasing the celiac trunk (C, black arrow). Axial T2-weighted imaging (D, white star) showing the mass in the body of the pancreas with non-visualization of the body and tail of the pancreas.

Out of 10 patients, four presented with complaints related to the pancreas. Out of four patients with complaints related to the pancreas, a work-up was done for insulin-dependent diabetes mellitus in a 19-year-old female patient (Figure 1), and CECT of the abdomen was done for acute pancreatitis (Figure 2), chronic pancreatitis (Figure 3), and carcinoma pancreas (Figure 4) in the rest of the three patients.

**Figure 5:**
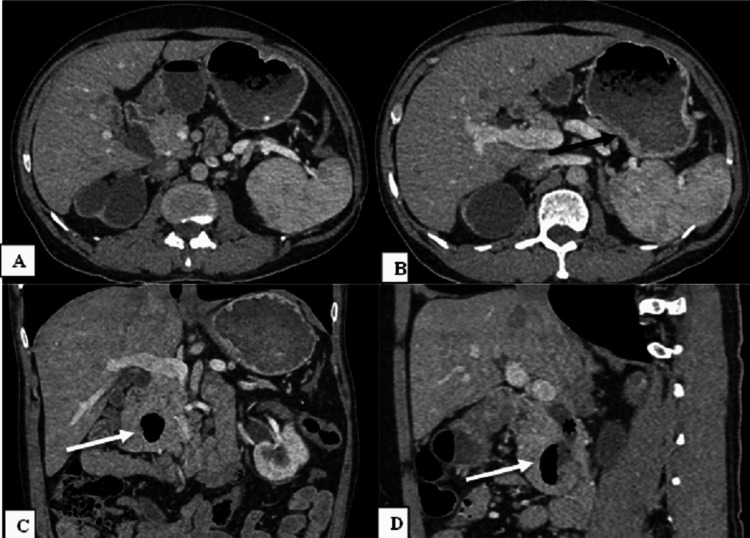
Partial dorsal agenesis of the pancreas. Axial contrast-enhanced computed tomography of the abdomen showing non-visualization of the body and tail of the pancreas (A) and dependent stomach sign (B, black arrow). Coronal contrast-enhanced computed tomography of the abdomen (C and D) showing Lemmel syndrome (dilatation of common bile duct (black star) due to duodenal diverticula (white arrows)).

Six patients presented with nonspecific complaints in which one patient presented with trauma, one for the work-up of carcinoma breast, one for the work-up of carcinoma ovary, one for follow-up for bilateral nephrolithiasis and jaundice (Figure 5), one for generalized body swelling, and one for chronic dull pain in the abdomen.

Out of 10 patients, five had a history of diabetes mellitus, seven patients had pain in the abdomen, one had acute pancreatitis, one had chronic pancreatitis, one had both chronic pancreatitis in the head and adenocarcinoma in the body of the pancreas, and three patients had jaundice. Out of 10 patients, four had complete dorsal agenesis of the pancreas and six had partial agenesis of the pancreas. All four patients with complete agenesis of the pancreas had a history of diabetes mellitus and only one out of six patients with partial agenesis of the pancreas had diabetes mellitus (Table 2). So, we can conclude that there is a strong association of diabetes mellitus in complete agenesis of the pancreas as compared to partial dorsal pancreatic agenesis with a statistically significant p-value (0.048).

**Table 2:** Strong association of diabetes in patients with complete agenesis of the dorsal pancreas. Chi-square statistical analysis revealed a statistically significant difference (p-value < 0.05) in the prevalence of diabetes mellitus between complete and partial agenesis of the dorsal pancreas.

Types	Diabetic	Non-diabetic	Total
Complete agenesis of the dorsal pancreas	4	0	4
Partial agenesis of the dorsal pancreas	1	5	6
	5	5	10

## Discussion

**Figure 6:**
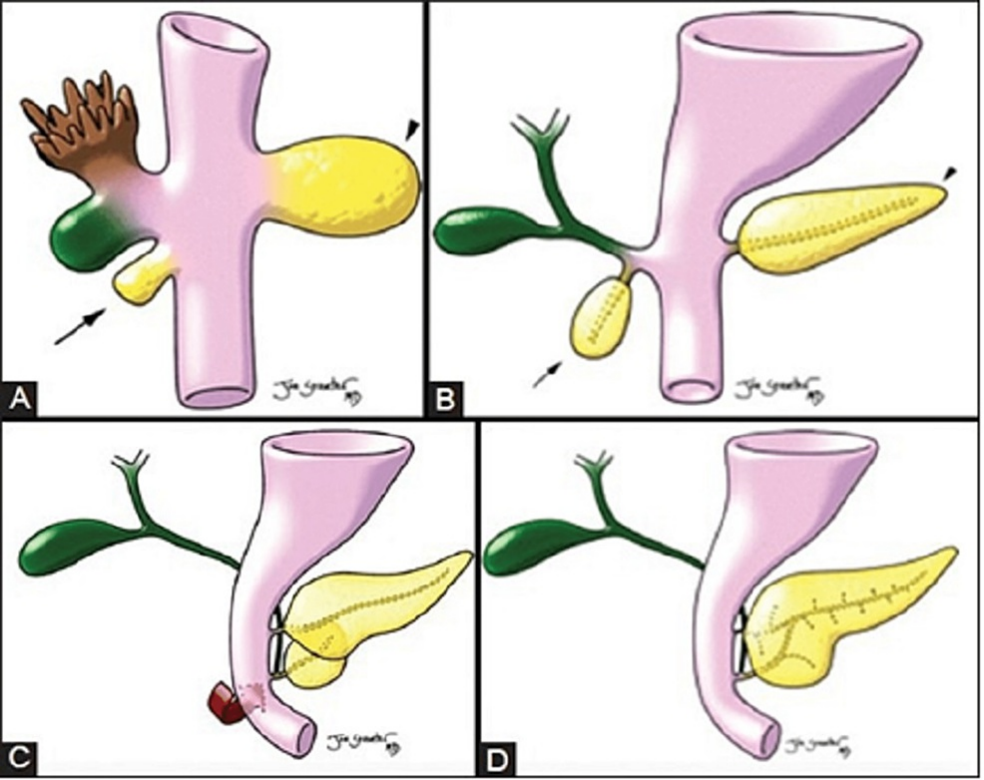
Schematic illustration of the normal embryological development of the pancreas. The hepatic diverticulum gives rise to the ventral pancreatic bud (arrows in A and B). The dorsal mesogastrium gives rise to the dorsal pancreatic bud (arrowheads in A and B). (C) The ventral bud rotates clockwise around the caudal part of the foregut, and the dorsal pancreas (located anteriorly) and ventral pancreas (located posteriorly) fuse. (D) Finally, the dorsal and ventral pancreatic ducts fuse, and the pancreas is drained primarily through the ventral duct. Copyright: © Indian Journal of Radiology and Imaging Source: Thakur S, Jhobta A, Sharma D, Thakur CS: MR in complete dorsal pancreatic agenesis: Case report and review of literature. Indian J Radiol Imaging. 2014, 24:156-9. 10.4103/0971-3026.134401. This is an open-access article distributed under the terms of the Creative Commons Attribution-Noncommercial-Share Alike 3.0 Unported, which permits unrestricted use, distribution, and reproduction in any medium, provided the original work is properly cited.

The pancreas develops from dorsal and ventral buds that appear as outgrowths of the primitive foregut in the fifth gestational week. The major part of the head and uncinate process are formed by the ventral bud, whereas the upper part of the head, body, and tail are formed by the dorsal bud. By the seventh gestational week, the ventral bud rotates and passes behind the duodenum from right to left, fusing with the dorsal bud [5]. Following this fusion, the ductal systems anastomose, which is a complex process with a wide range of possible outcomes. The duct of Wirsung is the portion of the ventral duct between the dorsal-ventral fusion and the major papilla. The main pancreatic duct is the portion of the dorsal duct that runs upstream to the dorsal-ventral fusion point. The duct of Santorini, or accessory pancreatic duct, drains at the minor papilla and is a segment of the dorsal duct downstream of the dorsal-ventral fusion point [5]. Figure 6 shows a schematic illustration of normal embryogenesis of the pancreas.

In complete dorsal agenesis, the structures developed from the dorsal bud, such as the neck, body, tail, minor papilla, and duct of Santorini, are absent. In partial agenesis, variable size of the body, a remnant of the accessory duct, and minor duodenal papilla are seen [6].

Dorsal pancreatic agenesis is an extremely rare congenital anomaly. The first case was reported in 1911 and was based on an autopsy. Only 106 such cases were reported between 1911 and 2015 [7]. The condition may exhibit autosomal dominant, X-linked dominant, or sporadic inheritance. It has been linked to extremely rare conditions such as heterotaxy and polysplenia. The close proximity of the developing pancreas to the spleen in the dorsal mesogastrium could explain this. It is also linked to congenital heart defects such as septal defects, tetralogy of Fallot, and pulmonary artery stenosis. The cause and mechanism of dorsal pancreatic agenesis are unknown. Changes in some signaling pathways (retinoic acid and hedgehog) have recently been shown to play a role. These signaling pathways have also been linked to pancreatic ductal adenocarcinoma pathogenesis [8].

The majority of agenesis of the dorsal pancreas patients are asymptomatic; however, when symptomatic, the majority of cases present with epigastric pain. In most cases, the pain is localized to the epigastric region and is exacerbated after eating [9]. This disease may also be associated with non-specific, persistent, and unexplained symptoms such as bloating or uncontrolled blood sugar [10]. Diabetes mellitus affects approximately 50% of those affected [5]. In our study, we saw a history of diabetes mellitus in five out of 10 cases. All four patients with complete agenesis had diabetes mellitus. Recurrent pain can be caused by a lack of papillary muscles or by concurrent acute or chronic pancreatitis [5], which can be caused by sphincter of Oddi dysfunction, compensatory enzyme hypersecretion, residual ventral gland hypertrophy, and elevated pancreatic intra-ductal pressure [11].

The majority of dorsal pancreatic agenesis patients develop recurrent or intermittent pancreatitis over time. There have been case reports in the literature that link pancreatitis to dorsal pancreas agenesis. Mohapatra et al. [4] presented a case of chronic calcifying pancreatitis that was comparable to ours. Cienfuegos et al. [12], in their systematic review, described the association of pancreatic neoplasm and non-alcoholic pancreatitis in the cases of agenesis of the dorsal pancreas. We also saw a patient with both biopsy-proven pancreatic adenocarcinoma and chronic pancreatitis in the residual pancreas in the dorsal pancreatic agenesis case. Acute pancreatitis was the finding we saw associated with one of our cases. Steatorrhea and signs of pancreatic exocrine failure were also reported by some patients [9].

The differential diagnosis of this entity includes pseudoagenesis, pancreatic head carcinoma, pancreatic divisum, pancreatic pseudolipodystrophy, pancreatic tumors, and distal pancreatic lipomatosis. With recent advancements in newer imaging techniques, diagnosis of agenesis of the dorsal pancreas appears to be a complete reality, and it is being diagnosed more frequently [9].

Although USG is the primary imaging study, the body and tail of the pancreas are inconspicuously visualized and frequently reported normally due to organ screen and overlying bowel gas shadows [10]. In MDCT, two useful signs (dependent stomach sign and dependent intestine sign) are described for differentiating distal pancreatic agenesis from other differentials such as pancreatic lipomatosis and pancreatic atrophy [3]. Abundant fat tissue is seen anterior to the splenic vein in cases of distal pancreatic lipomatosis. Similarly, fatty replacement is seen anterior to the splenic vein in pancreatic atrophy. In contrast, the stomach or intestine, which abuts the splenic vein, occupies the distal pancreatic bed in cases of distal pancreatic agenesis [13]. However, the ductal anatomy is not well delineated on CT. Therefore, MRCP or endoscopic retrograde cholangiopancreatography (ERCP) is required for further evaluation. MRI, including MRCP, is a noninvasive method that can clearly evaluate pancreatic ductal anatomy and aid in diagnostic confirmation. On MRI, T2WI should be used for interpretation as T1-weighted images of the pancreatic head and collapsed bowel loops in the distal pancreatic bed may show similar signals to the distal body and tail of the pancreas [8]. As ERCP is an operator-dependent invasive procedure requiring catheterization of minor papilla causing morbidity to the patient and carrying radiation exposure, it should be deferred and only used when there is ambiguity in radiological diagnosis [14].

There is no specific treatment mentioned in the literature and the patients are usually managed symptomatically.

## Conclusions

We conclude that though agenesis of the dorsal pancreas had been thought of as a rare congenital anomaly; however, increased use of cross-sectional imaging helps us detect this entity at a relatively higher rate as compared to before. Advanced MRI techniques like MRCP confirm the dorsal agenesis of the pancreas by demonstrating the pancreatic ductal anatomy. It ranges from partial to complete absence of the dorsal pancreas.

In our study, we found that diabetes mellitus is more prevalent in complete dorsal agenesis than partial agenesis. Also, insulin-dependent diabetes mellitus at a young age should prompt clinicians to rule out this rare possibility of dorsal agenesis of the pancreas. There can be a myriad of symptoms to no symptoms at all. The knowledge of the association of various clinical conditions with dorsal agenesis of the pancreas, such as non-specific abdomen pain or diabetes mellitus, will help the treating physicians in better management of the patients. There are no treatment guidelines mentioned in the literature and the patients are treated symptomatically.
